# Development of a 3D-printed, patient-specific stereotactic system for bihemispheric deep brain stimulation

**DOI:** 10.1186/s41205-023-00193-9

**Published:** 2023-10-13

**Authors:** Patrick Knorr, Dirk Winkler, Fabian Kropla, Robert Möbius, Marcel Müller, Sebastian Scholz, Ronny Grunert

**Affiliations:** 1 Department of Neurosurgery, University of Leipzig, Liebigstr. 20, 04103 Leipzig, Saxony, Germany; 2 The Medical Forge, Biosaxony, 04103 Leipzig, Saxony, Germany; 3 Fraunhofer Institute for Machine Tools and Forming Technology, 01187 Dresden, Saxony, Germany; 4 Fraunhofer Institute for Machine Tools and Forming Technology, 02763 Zittau, Saxony, Germany

**Keywords:** Deep brain stimulation, Parkinson’s disease, 3D printing, Patient-specific stereotaxy, Bihemispheric stimulation

## Abstract

The aim of the project was to develop a patient-specific stereotactic system that allows simultaneous and thus time-saving treatment of both cerebral hemispheres and that contains all spatial axes and can be used as a disposable product. Furthermore, the goal was to reduce the size and weight of the stereotactic system compared to conventional systems to keep the strain on the patient, who is awake during the operation, to a minimum. In addition, the currently mandatory computed tomography should be avoided in order not to expose the patient to harmful X-ray radiation as well as to eliminate errors in the fusion of CT and MRI data.

3D printing best meets the requirements in terms of size and weight: on the one hand, the use of plastic has considerable potential for weight reduction. On the other hand, the free choice of the individual components offers the possibility to optimize the size and shape of the stereotactic system and to adapt it to the individual circumstances while maintaining the same precision. The all-in-one stereotactic system was produced by means of the Multi Jet Fusion process. As a result, the components are highly precise, stable in use, lightweight and sterilizable. The number of individual components and interfaces, which in their interaction are potential sources of error, was significantly reduced. In addition, on-site manufacturing leads to faster availability of the system.

Within the project, a patient-specific stereotaxy system was developed, printed, and assembled, which enables the execution of deep brain stimulation via only three bone anchors located on the skull. Pre-developed MRI markers, which can be screwed directly onto the bone anchors via the sleeves, eliminate the need for a CT scan completely. The fusion of the data, which is no longer required, suggests an improvement in target accuracy.

## Introduction

 In course of demographic change and an ever-aging society, an increase in geriatric diseases such as tumors and Parkinson’s disease is to be expected [1]. Deep brain stimulation (DBS) can especially provide relief from movement disorders such as idiopathic Parkinson’s disease in addition to essential tremor, dystonia, compulsion, and depression [2–6]. Brain stimulation via targeted placement of electrodes can treat uncontrolled tremors, akinesia, and rigor as cardinal symptoms [7]. There are two commonly used targets for DBS, the subthalamic nucleus (STN) and the globus pallidus internus (GPi). The STN is cited as the favored target region for the stimulatory treatment of Parkinson’s disease [8–12].

Deep brain stimulation is a branch of functional stereotaxy. Stereotaxy describes a neurosurgical procedure that is characterized by image-guided and computer-assisted targeting systems to ensure the exact position of the patient in relation to the therapy device [13]. Every stereotactic surgery is based on the principle of a Cartesian coordinate system [14, 15]. In this process, a three-dimensional space is virtually built around the patient’s head using anatomical landmarks [7]. This allows us to clearly describe each point of the head and especially of the brain via Cartesian coordinates.

The systems that are currently used to perform deep brain stimulation can be divided into the following two main classes: conventional stereotactic frames, including an aiming arm device, e.g., the Leksell Stereotactic System (Elekta company), and new types of individually additively manufactured stereotactic platforms, e.g., the STarFix platforms (FHC company). The former refers to complexly designed stereotactic systems with large dimensions, a frame weighing over 2000 g and many additional assembly components. Since such systems are located on the patient’s head during imaging, targeting as well as complete intracranial surgery, there is a high level of strain on the patient. In addition, during surgery, patients are fixed to the operating table through the stereotactic unit, which is also considered a negative aspect [16, 17]. A list of advantages and disadvantages of both systems is shown in Table 1.

**Table 1:** Advantages and disadvantages of the stereotactic systems

conventional stereotactic frames including an aiming arm device	Patient-individually manufactured stereotactic platforms
**+** high stiffness **+** variable use **+** recognized state of the art	**+** low mass (platform about 250 g) **+** few individual components **+** easy to operate **+** platform as a single-use product ◊ no further sterilization processes
**–** large dimensions **–** large weight ◊ high discomfort to the patient **–** patient fixation to the operating table **–** very complex, many individual components **–** long training periods **–** effort and costs for maintenance **–** space for storage required **–** need to be reset for each target point	**–** can be used only for the two defined target points **–** long delivery times due to production in USA and subsequent delivery including import control (up to 3 weeks) ◊ daily clinic routine difficult to plan **–** Assembly of the feed units ◊ time-consuming, source of errors **–** low acceptance among neurosurgeons

With the use of both systems, patients are exposed to X-rays due to the mandatory computer tomography (CT) scan. Furthermore, the fusion of CT and magnetic resonance imaging (MRI) data holds additional potential for error.

In the following, we will describe the surgical procedure plastic system for bihemispheric deep brain stimulation from the FHC (STarFix Bilateral Platform). This system is currently only used in Germany at the Clinic and Polyclinic for Neurosurgery at Leipzig University Hospital and is one of the few systems thus far that enables simultaneous, bilateral stimulation. Prior to the actual surgery, four bone anchors are placed on the calvaria under local anesthesia and imaging is performed. Imaging includes both a cranial CT scan and a cranial MRI scan [7]. The CT scan is mainly used to visualize the bone anchors. With the MRI scan, the functional regions can be visualized, and the position of the electrodes can be planned in additional software. The placement of the bone anchors, including the required imaging, decouples the surgical preparation from the stereotactic surgery [18]. After the data sets have been merged, the entry and destination point coordinates are defined. Here, the fusion of the data sets poses a major problem in terms of accuracy. The two imaging methods have different coordinate systems and represent the anatomical structures differently in each case [19]. These coordinates, in combination with the bone anchor coordinates, provide the basis for designing the patient-specific stereotactic platform. The 3D-printed platform already contains all the spatial coordinates relevant for intracranial intervention. For the surgical procedure, the stereotactic platform is attached to the already implanted bone anchors with screws. This is followed by double-sided borehole trepanation and fixation of the electrode feed systems (each about 500 g) to the platform (about 250 g). After performing accuracy validation using a target point simulator, the intraoperative stimulation of the target areas began using test electrodes. The stimulation starts approximately 10 mm above the planned target point and ends approximately 2–5 mm below [20]. The motor responses are observed and at the point of greatest clinical effect, with absent or minimal side effects, the permanent electrodes are placed and fixed using miniplates and bone cement [21, 22]. Wound closure and removal of the bone anchors complete the surgery. A required pulse generator is implanted below the clavicle in the same session or after a few days. In addition to the advantages listed in Table 1, the reduction of inaccuracies due to motion artifacts and the simultaneous supply of both cerebral hemispheres can be mentioned as advantages of this system [16]. Another positive effect is the reduction of the duration for electrode placement by approximately one hour [2, 23].

## Materials and methods

Considering the high demands of medical technology, such as biocompatibility, steam sterilizability, high print quality and the possibility of color printing, the Multi Jet Fusion Technique (MJF) developed by HP was used to manufacture the patient-specific stereotactic system.

The MJF process offers numerous advantages compared to other additive manufacturing technologies, the most important of which are listed below [24]:

- functional colors.
- weight reduction.
- supervision of the process.
- biocompatible materials.
- Chemical resistance.
- high accuracy (layer thickness 0.08 mm).

The component accuracy to be achieved for the MJF process is specified as ± 0.3% (but at least ± 0.3 mm) [25]. In internal studies conducted previously, the influence of hot steam sterilization on the dimensional stability of components printed from PA12 was also investigated. For this purpose, the components (similar in shape and size to the stereotactic device) were subjected to sterilization according to EN ISO 13485:2016, and the geometries before and after sterilization were measured using CT. The deviations identified in the target/actual comparison were less than 0.1 mm on average. In an analysis carried out by HP regarding the influence of sterilization using holes of different sizes, deviations of less than 0.015 mm were found based on 3D scans [26].

**Fig. 1:**
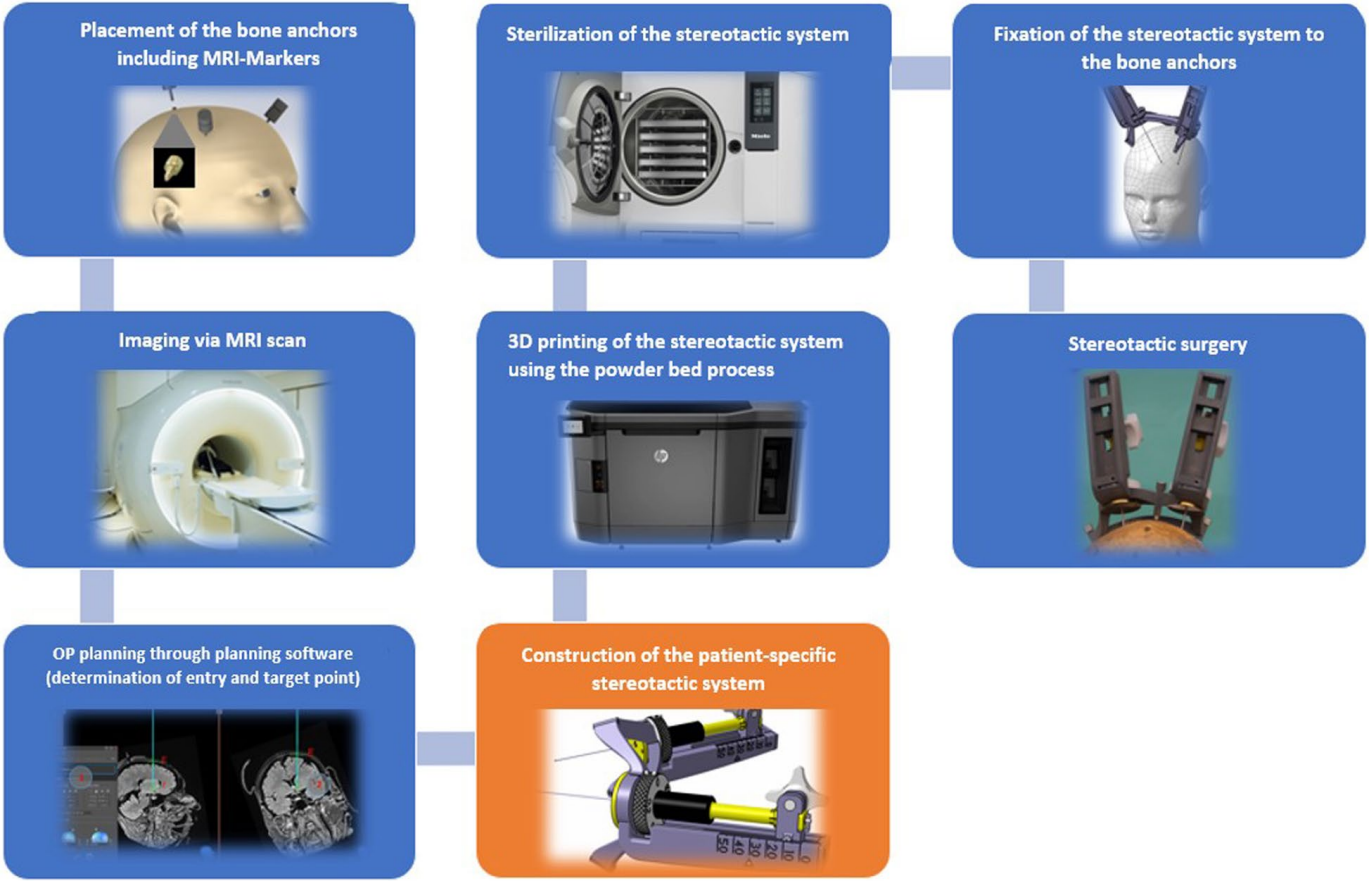
Workflow of the developed overall system for deep brain stimulation integrating CAD modeling [26–28]

The workflow (Fig. 1) serves to integrate the design of the stereotactic system into the overall system that has been developed. In this regard, imaging already differs from all currently used methods for image acquisition. The special in-house developed MRI markers (visible on MRI and CT), which can be screwed directly onto the bone anchors, allow imaging by MRI scan alone. A CT scan is not needed; therefore, the associated radiation exposure is eliminated. The mentioned MRI markers consist of a cylindric body and two axially arranged gel balls with diameter of 7 mm, inside. The gel balls are commercial vitamin d capsules type Dekristol 1000 I.E. (Mibe GmbH, Sandersdorf-Brehna, Germany). The alignment of the bone anchors can be clearly defined via the center points of the spheres. With the aid of our own software, which automatically recognizes the MRI markers, surgery planning and determination of the entry and target point coordinates are carried out. The desired target points are determined by the neurosurgeon and based on this; the trajectories are defined in such a way that there is no damage to critical brain areas. After construction, which is described in detail in the following, 3D printing of the stereotactic system including cleaning and sterilization, takes place. Immediately prior to the stereotactic procedure, the complete stereotactic system is fixed to the bone anchors using screws.

**Fig. 2:**
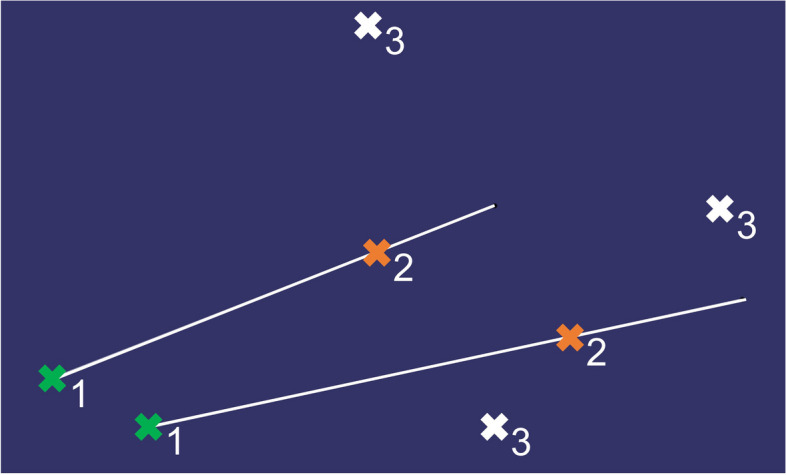
Base points of the construction: (1) target points, (2) entry points, (3) attachment points

The base of the construction are the two target points defined by Cartesian coordinates, the two entry points, and the three attachment points (Fig. 2). All these points must be provided in advance by a specialized neurosurgeon. The three attachment points prevent the system from tilting during the subsequent operation.

**Fig. 3:**
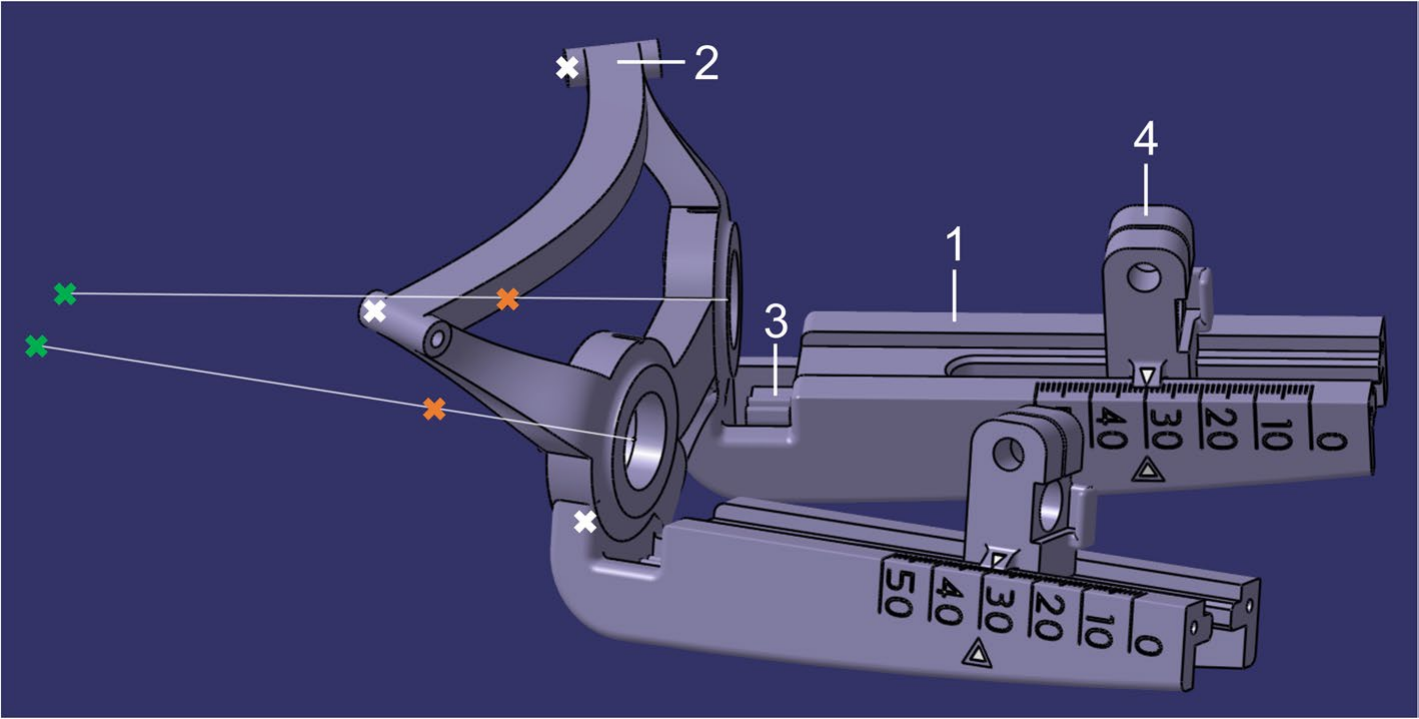
Stereotactic system including (1) feed units, (2) sleeves, (3) elastic elements and additionally (4) slides

The CAD design of the stereotactic system starts with three sleeves (Fig. 3), which are later used to fix the system to the implanted bone anchors. Subsequently, the electrode feedthroughs were constructed along the axes of the target and entry points at a defined distance to the skull. The distance was chosen in such a way that, on the one hand, accessibility for the surgeon is ensured and, on the other hand, the distance to each of the target points is identical. Sleeves and electrode feedthroughs were then connected by torsion-resistant, curved elements.

Since the feed units for both target points resemble each other, the explanation of the construction process is only given for one of them. The platform and the feed units are connected, to create a single printing component (Fig. 3).

**Fig. 4:**
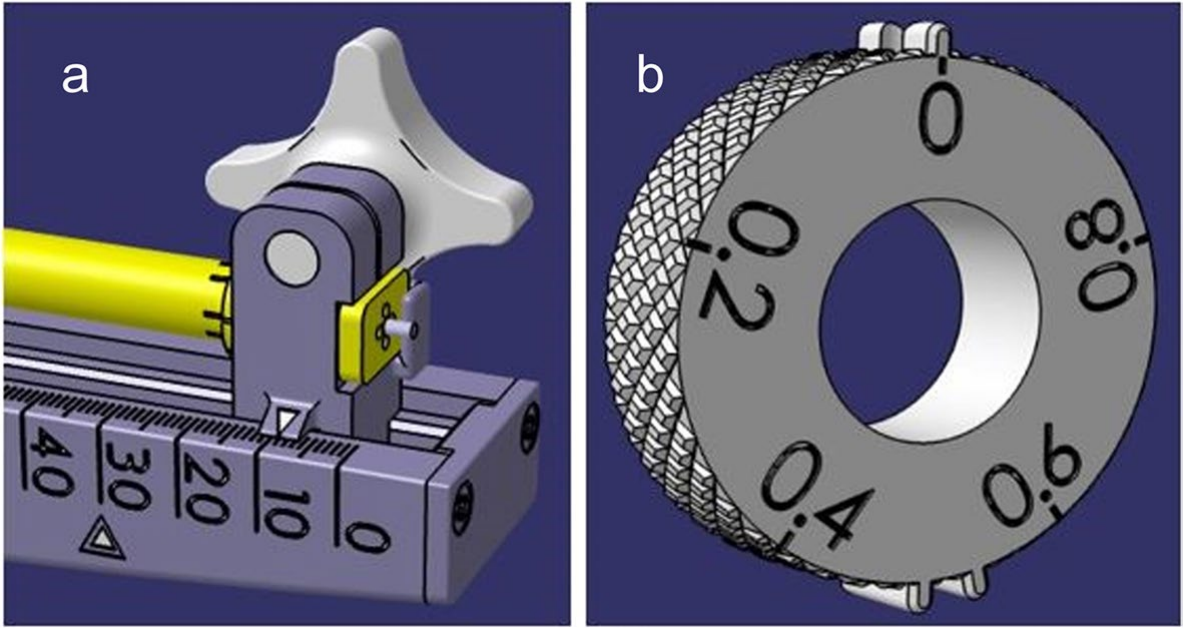
**a** Slide with arrow for reading on the depth scale, (**b**) Handwheel with knurled structure and fine adjustment

The feed unit serves as a guide for the linear feed of the electrodes. Furthermore, it contains a depth scale from 0 to 50 mm, the marking of the planned target point and a unique designation of the patient-specific system. An additional elastic element (Fig. 3) on the underside of the feed unit serves to provide a latching function. Subsequently, the slide was designed and adapted to the guide. Particular attention was given to a tight clearance fit. The slide also includes an arrow for indication on the depth scale (Fig. 4a), a holder for the stop plate and a printed thread for the cross handle. By screwing the cross handle, the slide is pressed together in the upper area, the guide sleeve is fixed, and the electrode guides, including electrodes, are clamped. The guide sleeve is designed so that up to five electrodes can be inserted through it. In the front area, the guide sleeve has a printed fine thread for precise feeding during use. The counterpart of this sleeve is a bushing with a collar, which has the corresponding internal thread. The collar provided on the bushing serves as a stop in the stereotactic system. The width and diameter of the collar were adapted to the geometry of the device to prevent slipping or tilting.

The handwheel (Fig. 4b) contains a bore whose diameter corresponds to the outer diameter of the bushing to allow it to be plugged on. The handwheel can be fixed to the bushing through a provided thread. This connection makes it possible to convert the rotational motion generated via the handwheel into translational motion. A knurled structure on the handwheel ensures safe operation. The handwheel also has three knobs, which, after assembly, engage with the latching function provided in the feed unit. In addition to the latching function, the handwheel has been provided with a scale division in 0.2 mm steps to guarantee fine adjustment along the z-axis. A guide plate is used to fix the bushing in the stereotactic system. The latter contains five holes (diameter of 1.85 mm) for the electrode guides, two holes for positioning and two holes for fixation. The guide plate as well as the stop plate were made of titanium to meet the high requirements in terms of accuracy. After assembly, the rear cover completes the design. Any fastening elements, such as countersunk and grub screws, are also made of titanium. The material titanium was chosen because of its biocompatibility and the possibility of color anodization.

**Fig. 5:**
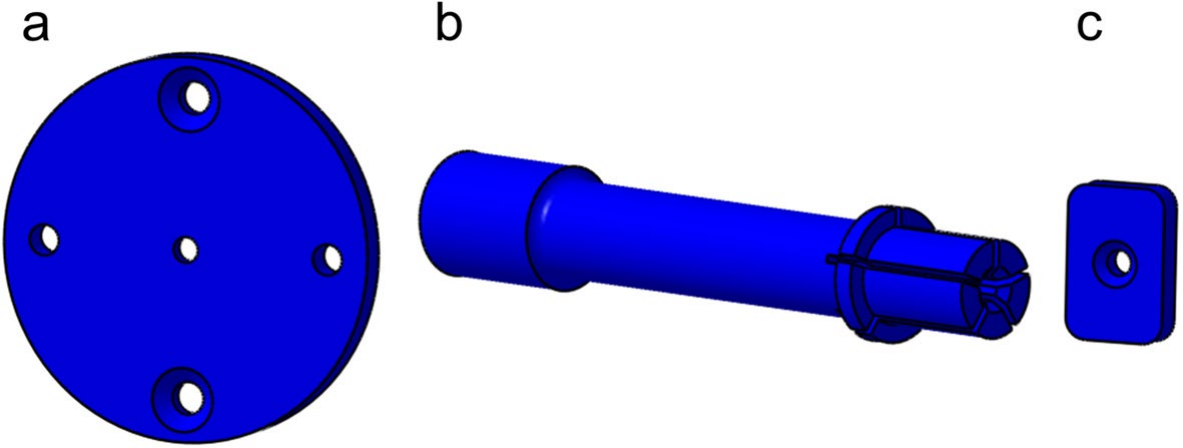
Elements for brain biopsy (**a**) guide plate, (**b**) guide sleeve, (**c**) stop plate

 The described product can be used for brain biopsy in addition to deep brain stimulation. For this purpose, three additional parts were developed, which can be easily exchanged. These parts are the guide plate, the guide sleeve, and the stop plate (Fig. 5). All of them are provided with only one hole (diameter of 2.5 mm) for guiding the biopsy needle.

To avoid confusion, the interchangeable components were printed in color or anodized. Blue indicates biopsy, and yellow indicates deep brain stimulation. To make the use as pleasant as possible for the surgeon, all scales were set off through colored printing, and the two components to be operated (handwheel, cross handle) were printed using white color.

## Results

**Fig. 6:**
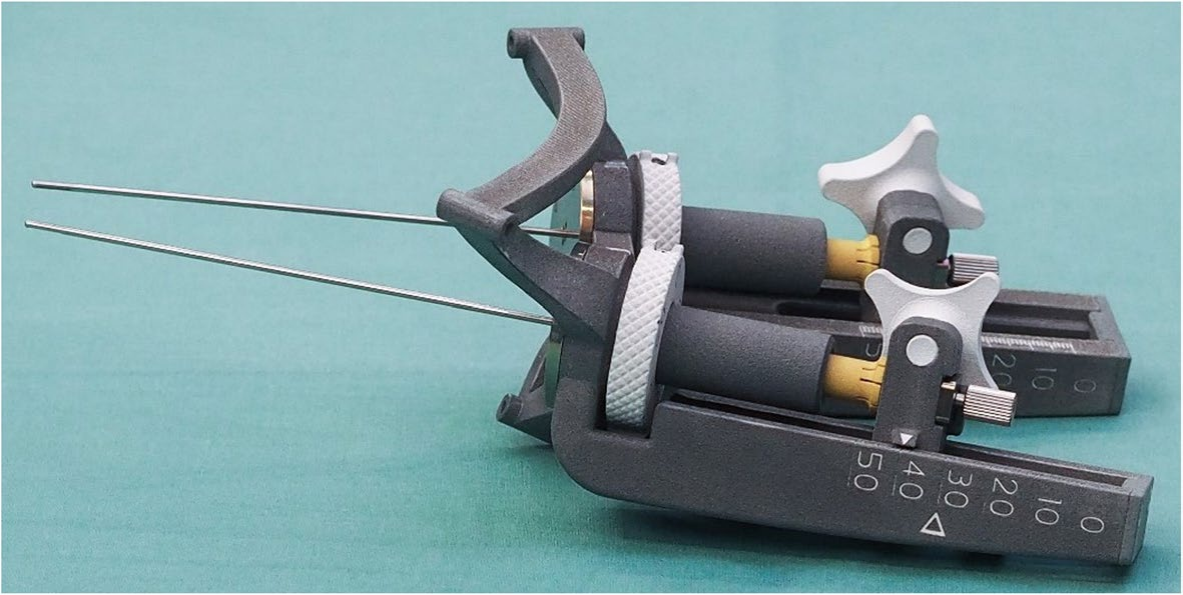
Preassembled stereotactic system for bihemispheric deep brain stimulation – Dimensions L x W x H 160 × 150 × 90 mm (without electrodes)

 The project has succeeded in combining the stereotactic platform and the two feed units in one piece. The spatial arrangement of the attachment points to the axes along which the electrodes are later moved is thus completed at an early stage. This is made possible by high-quality 3D printing using the MJF process. The PA12 material used in this process can be steam sterilized and thus meets the high clinical requirements. The possibility of full-color printing was applied to make use as easy as possible for the surgeon. In this way, the scales were highlighted with colors, the planned target point was marked, and the parts to be operated were printed in white. Because the system can also be used for biopsy as well as for deep brain stimulation, the respective components have been colored for identification. By exchanging just three components per feed unit, it is possible to switch from one treatment process to another. Yellow components indicate deep brain stimulation, and blue components indicate biopsy. By moving the electrodes forward via the handwheel and clamping the electrode guides via the cross handle, it was possible to implement simple handling and tool-free assembly. The click mechanism created via the handwheel, which characterizes full rotation with one click and half rotation with two clicks, contributes to user-friendliness. A grub screw located in the feed unit allows the user to adjust the prestressing of the latching function according to personal preference. The fine adjustment scale located on the handwheel allows the electrodes to be moved forward with an accuracy of 0.2 mm. The goal of reducing the number of individual components compared to conventional systems was also achieved. Thus, the design consists of 17 individual parts and 12 screws for assembly. Since the entire system can be provided to the user already fully assembled, there is no need for any preparation work. During the surgical intervention, only the electrode holders, including the electrodes, must be inserted and fixed. Since the manufactured system for bihemispheric deep brain stimulation was largely made of PA12, the total mass is only 230 g, which is only one fifth of the FHC system (1250 g). The considerable weight saving reduces the impact on the awake patient during surgery and the risk of a loss of accuracy due to gravity. Since the presented patient-specific stereotactic system (Fig. 6) is a single-use product, additional sterilization procedures after surgery are not needed.

## Discussion and conclusion

Compared to conventional stereotactic frames, the additively manufactured stereotactic system is characterized not only by considerable weight savings but also by the elimination of additional adjustments, for example, of angles. The reason for this is that the manufactured system already contains all the spatial coordinates required for the intracranial procedure. This fact, in combination with the placement of bone anchors performed on an outpatient basis followed by imaging, in analogy to the systems of FHC [16], enables the temporal decoupling of the surgical preparation from the stereotactic procedure. Compared to the systems from the FHC, the system introduced here does not require a CT scan at all. Therefore, there is no X-ray exposure for patients. Merging errors, which can occur when merging CT and MRI data, are also a thing of the past. In the stereotactic system presented here, it was possible to eliminate another interface (between the platform and electrode feed systems) and thus a potential source of error regarding accuracy. Since this eliminates the need to assemble the electrode feed systems, about 15 min of operating time can be saved. Furthermore, manufacturing of the new type of stereotactic system for bihemispheric deep brain stimulation, on an existing 3D printer could be done in the hospital, often eliminating significantly long delivery times, and thus simplifying the planning of surgery days. Completion of the 3D-printed system, including the corresponding assembly, shall take place within 48 h. The printing speed of the MJF process is up to 10 times faster than the SLS (selective laser sintering) process used by FHC [29]. The development is characterized by an easy-to-operate electrode feed via a handwheel as well as tool-free clamping of the electrode holders (including electrodes) by means of a cross handle. By reducing the complexity and simplifying the handling of the system, increased acceptance among neurosurgeons is expected. As a unique selling point, the invention offers the possibility to perform both deep brain stimulation and biopsy with only one system. The developed, patient-specific stereotactic system can be delivered to the user completely preassembled, thus avoiding long preparation and training times. To ensure the quality of all additively manufactured components before assembly, they are digitalized using 3D scans and compared with the target geometry.

To validate the development, an accuracy study will subsequently be conducted on body donors, the results of which will be part of an additional second publication paper. Accuracy in a first preliminary study ranged between 2.0 and 3.6 mm. This accuracy values are comparable to those of the CT based FHC system.

## Data Availability

All data generated during the process of development are included in this article. Further enquiries can be directed to the corresponding author.
